# The influence of pharmaceutical care in patients with advanced non-small-cell lung cancer receiving combination cytotoxic chemotherapy and PD-1/PD-L1 inhibitors

**DOI:** 10.3389/fphar.2022.910722

**Published:** 2022-10-18

**Authors:** Wen Kou, Yan Yan Lin, Fei Su, Yue Xiang, Hui Qiao, Xin’An Wu, Xiao-Ming Hou

**Affiliations:** ^1^ Department of Pharmacy, The First Hospital of Lanzhou University, Lanzhou, Gansu, China; ^2^ Department of General Surgery, First Hospital of Lanzhou University, Lanzhou, Gansu, China; ^3^ Department of Oncology, First Hospital of Lanzhou University, Lanzhou, Gansu, China; ^4^ United States Food and Drug Administration, Silver Spring, MD, United States

**Keywords:** pharmaceutical care, immune checkpoint inhibitors, immune-related adverse events, non-small-cell lung cancer, chemotherapy

## Abstract

**Background:** Immune checkpoint inhibitors combined chemotherapy (ICIC) are widely used for various types of lung cancer in the past decade. However, ICIC related adverse events (AEs) are more serious than immune-related adverse events (irAE) or cytotoxic chemotherapy alone.

**Objective:** This prospective interventional study aimed to evaluate the impact of the pharmaceutical care program in reducing adverse events and analyze pharmacy interventions in patients with NSCLC who receive ICIC therapies.

**Method:** NSCLC patients were enrolled in this study, the pharmaceutical care program was introduced after patients received the second cycle ICIC therapies, and were followed by the pharmacist for 6 months after hospital discharge. The percentages of adverse events between patients in and after the first two cycles were analyzed and compared.

**Results:** After the first two treatment cycles, the clinical pharmacist proposed 67 interventions in 30 patients. The most frequent types of intervention were drug discontinuation (40.3%, 27/67) followed by drug modification (14.9%, 10/67). There were significant decreases in AEs after the second cycle with respect to nausea (≥grade-2, 14% vs. 28.3%, *p* = 0.039), constipation (≥grade-2, 8.8% vs. 21.7%, *p* = 0.039), diarrhea (≥grade-2, 6% vs. 16.7%, *p* = 0.031), and myelosuppression (≥grade-2, 15.8% vs. 30.0%, *p* = 0.022).

**Conclusion:** Provision of pharmaceutical care for NSCLC patients receiving ICIC therapies can optimize drug therapy and reduce adverse events.

## Introduction

Lung cancer remained the leading cause of cancer-related death (Sung et al., 2021). Non-small cell lung cancer (NSCLC) contributes 75% of lung cancer, and most patients are diagnosed in the advanced stage. In 2020, lung cancer is the second most commonly diagnosed cancer (2.2 million) and 1.8 million deaths were estimated in the world (Sung et al., 2021). Platinum-based chemotherapy has been the standard for the first-line treatment of advanced NSCLC that lacks targetable driver mutations (Hanna et al., 2017). However, chemotherapy is associated with only modest efficacy and has reached a treatment plateau. Immune checkpoint inhibitors (ICI), including cytotoxic T-lymphocyte-associated protein 4 (CTLA-4), programmed cell death-*1* (PD-1), and programmed cell death ligand-1 (PD-L1) antibodies, are arguably the most important development in cancer therapy in the past decade. ICIs have been approved as first or second line treatment for various types of lung cancer (Remon et al., 2017). Chemotherapy has also been shown to induce PD-L1 expression in tumor cells. Several randomized controlled trials have shown that the combination of immunotherapy and chemotherapy synergistically improved PD-1 and PD-L1 monotherapies (Langer et al., 2016; Gandhi et al., 2018; Socinski et al., 2018). PD-1/PD-L1 inhibitor plus chemotherapy is statistically associated with a 38% reduction in the risk of disease progression, a 32% reduction in the risk of death, and 1.6 times the probability of achieving an objective response compared to standard chemotherapy for first-line treatment of advanced NSCLC (Zhou et al., 2018).

Despite impressive survival benefits with immunotherapy in patients with NSCLC, its use can be hampered by adverse events (AEs) related to excessive immune activation, collectively called immune-related adverse events (irAEs) (Chan et al., 2015). IrAEs often distinctly differ from the classical chemotherapy-related toxicities and potentially affect multiple organ systems. Although survival benefits are improving with the prevalence of combinatorial therapies of ICI and chemotherapy (ICIC), AEs can emerge simultaneously, posing new challenges for clinicians. ICIC therapies were significantly associated with a higher frequency of treatment-related AEs of grade 3 or more severity (pooled relative risk RR 1.14, 95% confidence interval CI 1.04–1.26, *p* = 0.007), AEs leading to treatment discontinuation (pooled RR 1.29, 95% CI 1.01–1.60, *p* = 0.022), serious AEs (pooled RR 1.70, 95% CI 1.17–2.49, *p* = 0.006), and immune-mediated AEs of any grade (pooled RR 2.37, 95% CI 1.98–2.84, *p* < 0.001) and of grade 3 or more severity (pooled RR 3.71, 95% CI 2.63–5.24, *p* < 0 .001) (Chan et al., 2015). Patients should be regularly monitored by a multidisciplinary team, ideally using a personalized surveillance strategy.

Pharmacists play an essential role in delivering care to cancer patients (Inoue, 2004; Aimono et al., 2013; Arakawa-Todo et al., 2013; Kimura et al., 2017; Tanaka et al., 2018; Todo et al., 2019; Lau et al., 2020). Pharmacists are involved in all phases of cancer treatment, from assessment and diagnosis to treatment decisions, medication management, symptom management, supportive care, and finally with survivorship programs at the end of treatment. Pharmacists work with other healthcare providers to ensure a current and accurate medication list, select the most appropriate therapy, monitor the effects of prescribed medications, and manage AEs. Several studies have evaluated pharmacist interventions in patients receiving ICIs and their impact on patient outcomes. In a Canadian study involving 143 patients initiated with ICIs, intensive education about irAEs, proactive follow-up, and management of irAEs reduced the odds of discontinuation of treatment due to irAEs (Myers et al., 2021). A study conducted in the United States described a pharmacist-driven program called the immune checkpoint inhibitor program. The program’s objective was to ensure patient and caregiver education and continuous monitoring of irAEs. The program led to earlier recognition and treatment of irAEs (Renna et al., 2019). Another study evaluated the effectiveness of a pharmacy consult service in identifying and managing irAEs in a large community hospital in the US. The pharmacy consultation service increased the identification of patients receiving ICI and led to timely interventions to manage irAEs. Interventions included the initiation/adjustment of steroid therapy, the placement of a consult for oncology or other specialists, and other therapeutic interventions (Kamta et al., 2021). Finally, a study evaluated the impact of a pharmacist-managed irAEs protocol in an oncology clinic in the United States. During the pilot period, 17 patients on ICIs were involved, pharmacists initiated 21 new medications to treat irAEs, including thyroid hormone replacement in 7 patients (41%) and oral corticosteroids in 6 patients (35%) with a total of 28 dose adjustments. Furthermore, the study showed a reduced number of physician hours per month to treat irAE, increased physician confidence in the management of irAE, and a desire for continued pharmacist management of irAE (Le et al., 2021).

These studies were conducted outside China, and there are few reports on pharmaceutical care in patients receiving ICIC therapies. In July 2020, a pilot pharmacist-managed pharmaceutical care program was launched at the First Hospital of Lanzhou University. The program targeted NSCLC patients receiving ICIC therapies, and this report described the effect of the pharmaceutical care program.

## Aim of the study

The study aimed to evaluate the impact of the pharmaceutical care program in reducing adverse events and analyze pharmacy interventions in patients with NSCLC who receive ICIC therapies.

## Materials and methods

### Setting and study population

The study was performed in the oncology department from July 2020 to March 2021 at the First Hospital of Lanzhou University, a 2,686-bed academic teaching hospital in Gansu province, northwest China. The oncology department has an inpatient unit and an outpatient clinic. The study inclusion criteria were patients diagnosed with NSCLC and received six cycles of ICIC treatment during hospital stay. Patients who discontinued treatment, transferred to other hospitals, could not complete the expected 6-month follow-up, or died during the duration of therapy were excluded.

### Study design

The study was a prospective interventional study divided into two phases. Phase one was when NSCLC patients received two cycles of ICIC therapies without the participation of pharmacists. The pharmaceutical care program was introduced before patients received the third cycle ICIC therapies. The patients received pharmacist care during treatment cycles 3–6 and were followed by the pharmacist for 6 months after hospital discharge (phase two). The percentages of AEs between patients in these two phases were analyzed and compared.

### The components of the pharmaceutical care program

The following were the components of the pilot pharmaceutical care program:

1) Identify concomitant non-chemotherapy drugs that could affect the efficacy of ICIC therapies. After the patients completed the second cycle treatment, the clinical pharmacist conducted medication reconciliation through patient interviews and checking the electronic medical record. Patients were asked about taking over-the-counter and other concomitant medications during the first two-cycle treatment. Drugs that could interact with the immunosuppressive drugs PD-1 or PD-L1 or affect the overall effect of ICIC therapies were identified. Before starting the third cycle of treatment, physicians and patients proposed recommendations to discontinue or adjust these concomitant drugs.
2) Reduce the incidence of AEs from ICIC therapies. In phase 2, all patients received pharmaceutical care from the clinical pharmacist. Pharmaceutical care activities included participating in multidisciplinary ward rounds, reviewing prescriptions, monitoring AEs, promptly following laboratory tests, providing drug information to physicians and nurses, and drug consultation for patients and their caregivers.
3) Provide psychological counseling and support to patients and caregivers in managing drug therapies and AE to build the treatment confidence of patients and their caregivers.
4) Conduct regular follow-up of patients for 6 months after discharge. The same pharmacist who provided inpatient care conducted patient follow-up. Each patient was scheduled for an outpatient clinic visit per month. A physician saw the patient, and a follow-up form was completed. The form was forwarded to the pharmacist to assess AEs. The pharmacist followed each patient twice a month through phone calls or WeChat communication (China’s largest social media platform), the follow-up contents focused on signs or symptoms that suggest AE, like ask patients if they exhibit a disrupted gait/dyspnea, dry cough, wheezing, tachy-cardia/or headache, fatigue, visual defects, these symptom might suggest patients are undergoing some AEs, like arthralgias/pneumonitis/hypophysitis. During the follow-up, patients were always be informed that irAEs can arise at any time during therapy, even long after treatment has stopped and were instructed to contact the pharmacist if they experienced any AE or signs and symptoms suggesting AE. The occurrence of AE was communicated to physicians to provide timely symptomatic treatment after discharge.
5) Provide non-pharmacological recommendations. These included: 1) lifestyle modification: for example, patients with bone marrow suppression after chemotherapy should avoid going to public places after discharge and wear masks to prevent infection when in public, and patients with a rash should wear loose cotton clothing to prevent aggravating itching; 2) diet: for example, avoid spicy and irritating food in patients developing gastrointestinal disorders after chemotherapy, 3) the importance of attending regular clinic follow-ups: for example, some adverse reactions can occur several months or even 6 months after discharge, and it is also necessary to check tumor progression; and 4) medication adherence, for example, patients should continue taking levothyroxine or corticosteroids due to immunosuppressive-induced hypothyroidism or pneumonia.

### Data collection and statistical analysis

The following demographic and clinical information was collected: sex, age, weight, height, Eastern Oncology Cooperative Group (ECOG) performance status, number of metastatic sites, types of chemotherapy, PD-1/PD-L1 inhibitors, and concomitant nonchemotherapy drugs. The severity of AEs was classified into < grade 2 and ≥ grade 2 based on the Common Terminology Criteria for Adverse Events (CTCAE) v5.0 (CTCAE v5.0, 2017).

All baseline and 6-month data collection and evaluation were performed by a clinical pharmacist who was not involved in the pharmaceutical care interventions. The two pharmacists had completed the certified standardized clinical pharmacist training by the China National Health Commission and had more than 10 years of hospital work experience. The two pharmacists received study protocol training, standardized data collection tools, and patient interview guides to ensure program fidelity.

A descriptive analysis of the patient’s demographic and clinical characteristics and the incidence of AEs was performed. Means were calculated for quantitative variables. Frequencies and percentages were calculated for categorical variables. The McNemar test was used to compare the AE variables. A *p*-value < 0.05 was considered statistically significant. All analyses were performed using SPSS 15.0.

## Results

### Patients

Thirty patients met the study inclusion criteria and were included. Table 1 shows the demographic and clinical characteristics of these patients. The median age was 65.3 years, and 70% (*n* = 21) were men. Twenty-seven (90%) patients had ECOG scores of 0–1. Most of the patients, 73.3% (22/30), had ≤2 metastatic sites. The most frequently prescribed PD-1 inhibitors were sintilimab (63.3%). A total of 76.7% (23/30) of the patients were taking non-chemotherapy drugs before cancer treatment: 13 patients (43.3%) on proton pump inhibitors (PPI, Omeprazole, Rabeprazole, Esomeprazole) or H_2_-antagonists (Cimetidine, Ranitidine), 12 (40%) on non-steroidal anti-inflammatory drugs (NSAID, Ibuprofen, Celecoxib, Meloxicam), 8 (26.7%) on antibiotics (Potassium amoxicillin clavulanate, Cefazolin), and 6 (20%) on steroids (Prednisone, Dexamethasone, Methylprednisolone).

**TABLE 1:** The demographic and clinical characteristics of patients (*n* = 30).

	*n* (%)
Age (years)	
Median (range)	65.3 (35–78)
Height (cm)	
Median (range)	172 (158–180)
Sex	
Male	21 (70%)
Female	9 (30%)
Weight (kg)	
<60	11 (36.7%)
≥60	19 (63.3%)
ECOG score	
0–1	27 (90%)
2	3 (10%)
Number of metastatic sites	
<2	22 (73.3%)
≥2	8 (26.7%)
Type of chemotherapy and anti-PD-1/PD-L1 agent	
Cisplatin + pemetrexed + camrelizumab	7 (23.3%)
Cisplatin + pemetrexed + sintilimab	6 (20.0%)
Carboplatin + docetaxel + sintilimab	4 (13.3%)
Carboplatin + gemcitabine + sintilimab	4 (13.3%)
Carboplatin + paclitaxel + sintilimab	5 (16.7%)
Carboplatin + paclitaxel + Toripalimab	4 (13.3%)
Patients taking concomitant drugs in phase-1 cycles treatment	
Steroids	6 (20.0%)
Antibiotics	8 (26.7%)
Proton Pump inhibitors	8 (26.7%)
H_2-_antagonists	5 (16.7%)
NSAIDs	12 (40.0%)

ECOG, eastern oncology cooperative group; PD-1, program cell death protein 1; PD-L1, programmed cell death ligand 1; NSAID, nonsteroidal anti-inflammatory drugs.

### The analysis of concomitant medications after two cycles of cancer treatment

**FIGURE 1:**
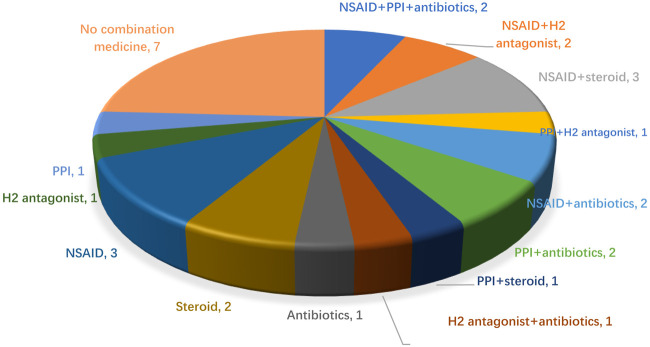
Patient concomitant medications before the third chemotherapy cycle.

After the patients received two cycles of ICIC therapies, medication reconciliation was performed, six patients (20%, 6/30) took steroids, and four took them for cancer-related symptoms. Eight patients (26.7%, 8/30) took antibiotics, 3 (10%, 3/30) took them prophylactically to prevent pulmonary infections, and the other patients took them (non-regularly) for possible infection symptoms such as coughing and difficulty breathing. Thirteen patients (43.3%, 13/30) took prophylactic gastric acid suppressants (8 on PPIs and 5 on H_2_-antagonists). Twelve patients (40%, 12/20) took NSAIDs for pain relief. In patients taking concomitant drugs, 12 (40%, 12/30) were taking two drugs, 2 (6.7%, 2/30) were taking three drugs (NSAID, PPI, and antibiotics), and 9 (30%, 9/30) were taking one drug, the specific medication before starting ICIC is shown in Figure 1.

### The pharmacist interventions in phase two

After the first two treatment cycles, the clinical pharmacist proposed 67 interventions in 30 patients (Table 2). The most frequent types of intervention were drug discontinuation (40.3%, 27/67) followed by drug modification (14.9%, 10/67). PPIs or H_2_-antagonists (48.1%, 13/27) were the most common discontinued drugs. Within the drug modification category, the most common drug modifications due to drug-drug interactions (DDI) were platinum-taxane (30%, 3/10), metoclopramide-selective serotonin reuptake inhibitors (30%, 3/10), and corticosteroids-NSAIDs (30%, 3/10). Adjusting the dose of granulocyte colony-stimulating factor G-CSF (53.8%, 7/13) was the most common intervention in the drug dose adjustment category (19.4%, 13/67). Adding antiemetic drugs (35.3%, 6/17), levothyroxine (23.5%, 4/17), and hydration (23.5%, 4/17) accounted for the most within the need of additional drug therapy category (25.4%, 17/67). Physicians accepted 65 (97%) of these interventions.

**TABLE 2:** Interventions after the second-cycle of chemotherapy.

Category of intervention (*n* = 67)	Contents of intervention (*n*, %)
Drug discontinuation (27, 40.3%)	Steroids (6, 9.0%) antibiotics (8, 11.9%) PPI or H_2_ antagonist (13, 19.4%)
Drug modification (10, 14.9%)	Platinum-taxane (3, 4.5%), NSAIDs-pemetrexed (2, 3.0%) metoclopramide-SSRIs (3, 4.5%) corticosteroids-NSAID (3, 4.5%) NSAID-SSRIs (2, 3.0%)
Drug dose adjustment (13, 19.4%)	G-CSF (7, 10.4%) other drugs (3, 4.5%)
Drug addition (17, 25.4%)	Antiemetic drugs (6, 9.0%) vitamin B_12_ or folic acid on pemetrexed chemotherapy (3, 4.5%) hydration on cisplatin chemotherapy or renal dysfunction (4, 6.0%) levothyroxine for the possibility of adrenal insufficiency (4,6.0%)

PPI, proton pump inhibitor; G-CSF, Granulocyte colony-stimulating factor.

### The analysis of adverse events during cancer treatment

The occurrences of AE were analyzed after each treatment cycle (Table 3). The most common AEs in phase 1 were myelosupression (85%), nausea (51.6%), and constipation (43.4%); and nausea (48.1%), myelosupression (40.9%), and vomiting (29.8%) in phase 2. In terms of AE severities, in phase 1, the top three AEs (grade <2) were myelosuppression (55%), nausea (23.3%) and constipation (21.7%); and the three AEs (grade ≥2) were myelosupression (30%), nausea (28.3%) and constipation (21.7%). In phase 2, the top three AEs (grade <2) were nausea (34.1%), myelosupression (25.1%), and thyroid dysfunction (18.5%); and the top three AEs (grade ≥2) were vomiting (15.8%), myelosupression (15.8%), and nausea (14%).

**TABLE 3:** The percentages of adverse events during each chemotherapy course.

Adr	Severity	Course1 (n, %)	Course2 (*n*, %)	Mean^1^ (%)	Course3 (*n*, %)	Course4 (*n*, %)	Course5 (*n*, %)	Course6 (*n*, %)	Mean^2^ (%)	*p* Value
Nausea	<Grade2	6 (20.0)	8 (26.7)	23.3	12 (40.0)	10 (34.5)	9 (32.1)	8 (29.6)	34.1	0.096
	≥Grade2	10 (33.3)	7 (23.3)	28.3	5 (16.7)	3 (10.3)	5 (17.9)	3 (11.1)	14.0	**0.039**
Vomiting	<Grade2	5 (16.7)	6 (20.0)	18.3	4 (18.3)	5 (17.2)	4 (14.3)	3 (11.1)	14.0	0.359
	≥Grade2	6 (20.0)	4 (13.3)	16.7	5 (16.7)	4 (13.8)	5 (17.9)	4 (14.8)	15.8	0.481
Constipation	<Grade2	8 (26.7)	5 (16.7)	21.7	5 (16.7)	4 (13.8)	3 (10.7)	2 (7.4)	12.2	0.118
	≥Grade2	6 (20.0)	7 (23.3)	21.7	3 (10.0)	2 (6.9)	3 (10.7)	2 (7.4)	8.8	**0.039**
Diarrhea	<Grade2	4 (13.3)	6 (20.0)	16.7	3 (10.0)	2 (6.9)	1 (3.6)	1 (3.7)	6.0	0.092
	≥Grade2	3 (10.0)	2 (6.7)	8.3	1 (3.3)	2 (6.9)	2 (7.1)	2 (7.4)	6.2	**0.031**
Myelosuppression	<Grade2	17 (56.7)	16 (53.3)	55.0	13 (43.3)	6 (20.7)	5 (17.9)	5 (18.5)	25.1	1.000
	≥Grade2	8 (26.7)	10 (33.3)	30.0	5 (16.7)	5 (17.2)	4 (14.3)	4 (14.8)	15.8	**0.022**
Pneumonitis	<Grade2	1 (3.3)	0 (0.0)	1.67	1 (3.3)	2 (6.9)	1 (3.6)	2 (7.4)	5.3	0.219
	≥Grade2	0 (0.0)	1 (3.3)	1.67	0 (0.0)	0 (0.0)	0 (0.0)	1 (3.7)	0.93	1.000
Arthralgias	<Grade2	3 (10.0)	2 (6.7)	8.3	3 (10.0)	2 (6.9)	1 (3.6)	1 (3.7)	6.0	1.000
	≥Grade2	1 (3.3)	1 (3.3)	3.3	0 (0.0)	1 (3.5)	2 (7.1)	1 (3.7)	3.6	0.688
Hepatotoxicity	<Grade2	3 (10.0)	4 (13.30)	11.7	2 (6.7)	2 (6.9)	3 (10.7)	4 (14.8)	9.7	0.607
	≥Grade2	1 (3.3)	2 (6.7)	5.0	1 (3.3)	1 (3.4)	2 (7.1)	2 (7.4)	5.3	0.508
Thyroid dysfunction	<Grade2	3 (10.0)	6 (20.0)	11.7	5 (15.0)	6 (16.7)	4 (20.7)	5 (14.3)	18.5	0.227
	≥Grade2	2 (6.7)	4 (13.3)	5.0	3 (10.0)	2 (10.0)	2 (6.9)	1 (7.1)	3.7	0.754

Mean^1^: the average incidence of adverse events during the first two courses of therapy.

Mean^2^: the average incidence of adverse events during the last four courses of therapy.

Bold values in column *p* value means *p* < 0.05.

Compared to phase 1, there were significant decreases in AE in phase 2 with respect to nausea (≥grade−2, 14% vs. 28.3%, *p* = 0.039), constipation (≥grade−2, 8.8% vs. 21.7%, *p* = 0.039), diarrhea (≥grade−2, 6% vs. 16.7%, *p* = 0.031), and myelosuppression (≥grade−2, 15.8% vs. 30.0%, *p* = 0.022).

### The analysis of non-pharmacological interventions during followup

**FIGURE 2:**
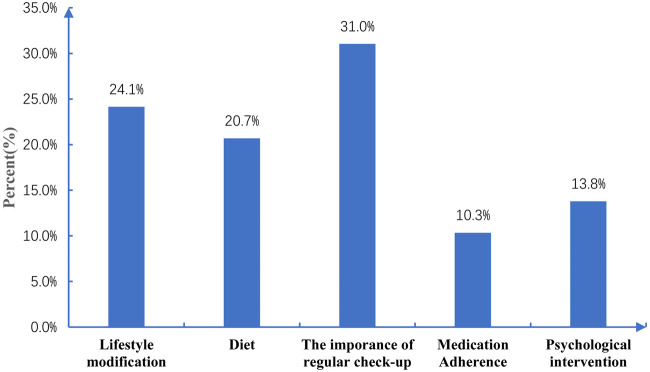
Percentages of non-pharmacological recommendations for patients.

The pharmacist proposed a total of 58 nonpharmacological recommendations to patients during the 6-month follow-up (Figure 2). The top three were the importance of engaging in regular clinic checkups (31%), lifestyle modification (24.1%), and diet (20.7%).

## Discussion

To our knowledge, our study is the first to describe the impact of a pharmaceutical care program in patients with NSCLC who receive ICIC therapies. The program can optimize patient drug therapies and reduce adverse events. In our study, almost 70% of the patients took systemic antibiotics and acid suppressants, and pharmacists proposed almost 30% of the interventions to discontinue these drugs. The impact of concomitant medications on PD-1 and PD-L1 remained elusive. Recent analysis shows that the gut microorganism can affect tumor-host interaction and plays a role in the therapeutic outcome of ICI (Havel et al., 2019). Both antibiotics and PPI can change the gut microbiome. PPI promotes hypochlorhydria, which reduces microbe diversity by increasing nitrate and bacterial nitrite reductase (Jackson et al., 2016). Antibiotics can alter the intestinal microbial environment and interfere with the response of T cells by altering cytokine production and dendritic cell activity (Hill et al., 2010). Currently, it is not conclusive that PPI use may affect the survival outcome of patients who receive agents that target the PD-1/PD-L1 pathway (Nguyen et al., 2019). However, several studies have indicated that antibiotic treatment is associated with a worsening of the clinical outcomes of PD-1 in cancer treatment, including lung cancer (Derosa et al., 2018; Huemer et al., 2018; Zhao et al., 2019). Pharmacists should assess the indication and duration of antibiotics and acid suppressants in ICIC therapies to optimize therapies.

Almost 20% of patients who receive PD-1/PD-L1 inhibitors have thyroid dysfunction, which usually occurs early in the course of treatment with a median onset of 6 weeks after drug initiation (de Filette et al., 2016; Lee et al., 2017). Thyroid hormone replacement should be initiated in patients with persistent hypothyroidism. In the US study involving 17 patients on ICIs, pharmacists proposed the addition of thyroid hormone replacement in 7 patients (41%) (Le et al., 2021). In our study, the pharmacist recommended the addition of levothyroxine in 13.3% of patients on ICIC therapies.

Drug modification due to DDI was the third common type of pharmacist intervention. For example, three patients were treated with platinum and paclitaxel chemotherapy simultaneously. Because platinum is likely to aggravate the myelosuppressive toxicity of taxane drugs, the pharmacist suggested changing the order of drug administration. The US FDA in 2020 published a draft guide on assessing DDI with therapeutic proteins (U.S. FDA, 2020). Although most PD-1/PD-L1 inhibitors are large molecules not commonly metabolized by cytochrome P450 (CYP450) enzymes, they may cause a transient release of cytokines, which may suppress the activity of the CYP450 enzyme leading to potential DDI. The DDI potential of pembrolizumab and atezolizumab has not been evaluated, and it is unknown whether they may modulate CYP450 activity by changing cytokines in the systemic circulation. Pharmacists should closely monitor patients receiving CYP450 substrates with a narrow therapeutic index and adjust the dose if necessary.

Similar to the other studies demonstrating the impact of pharmacy services in managing AEs, the pharmaceutical care program we initiated reduced the overall occurrences of AEs (Renna et al., 2019; Kamta et al., 2021; Le et al., 2021; Myers et al., 2021). Since ICIC therapies are relatively new, patients and their caregivers are not familiar with the medications, such as the therapeutic effects and the potential AEs, creating psychological anxieties and stress. In formulating the patient’s pharmaceutical care plan, the clinical pharmacist should educate patients and caregivers about the symptoms, onset, and duration of AE. For example, thyroid dysfunction induced by ICIC may not appear until after the completion of treatment. Management strategies should also minimize the discomforts of AEs and their impact on the continuation of treatment.

Our study has the following limitations: 1) the sample size was small with only 6 months of follow-up, 2) this was a single-center study, and the results may not apply to other settings, and 3) we did not evaluate the clinical results of pharmacist interventions.

## Conclusion

ICIC therapies are likely to impose more adverse react to patients, provision of pharmaceutical care especially medication reconciliation for NSCLC patients receiving ICIC therapies can optimize drug therapy and reduce adverse events. More studies are needed to evaluate the impact of the pharmaceutical care program on the clinical, economic, and humanistic outcomes in this population.

## Data Availability

The raw data supporting the conclusions of this article will be made available by the authors, without undue reservation.
